# CRISPR/Cas9-based Genome Editing in *Pseudomonas aeruginosa* and Cytidine Deaminase-Mediated Base Editing in *Pseudomonas* Species

**DOI:** 10.1016/j.isci.2018.07.024

**Published:** 2018-08-01

**Authors:** Weizhong Chen, Ya Zhang, Yifei Zhang, Yishuang Pi, Tongnian Gu, Liqiang Song, Yu Wang, Quanjiang Ji

**Affiliations:** 1 School of Physical Science and Technology, ShanghaiTech University, Shanghai 201210, China

**Keywords:** Genetics, Microbial Genetics, Biotechnology, Genetic Engineering

## Abstract

*Pseudomonas* species are a large class of gram-negative bacteria that exhibit significant biomedical, ecological, and industrial importance. Despite the extensive research and wide applications, genetic manipulation in *Pseudomonas* species, in particular in the major human pathogen *Pseudomonas aeruginosa*, remains a laborious endeavor. Here we report the development of a genome editing method pCasPA/pACRISPR by harnessing the CRISPR/Cas9 and the phage λ-Red recombination systems. The method allows for efficient and scarless genetic manipulation in *P*. *aeruginosa*. By engineering the fusion of the cytidine deaminase APOBEC1 and the Cas9 nickase, we further develop a base editing system pnCasPA-BEC, which enables highly efficient gene inactivation and point mutations in a variety of *Pseudomonas* species, such as *P*. *aeruginosa*, *Pseudomonas putida*, *Pseudomonas fluorescens*, and *Pseudomonas syringae*. Application of the two genome editing methods will dramatically accelerate a wide variety of investigations, such as bacterial physiology study, drug target exploration, and metabolic engineering.

## Highlights

• The pCasPA/pACRISPR system enables efficient genetic manipulation in *P*. *aeruginosa*
• The pnCasPA-EBC system allows rapid C→T base editing in *Pseudomonas* species
• The two tools will accelerate a variety of investigations in *Pseudomonas* species

## Graphical Abstract

**Figure:**
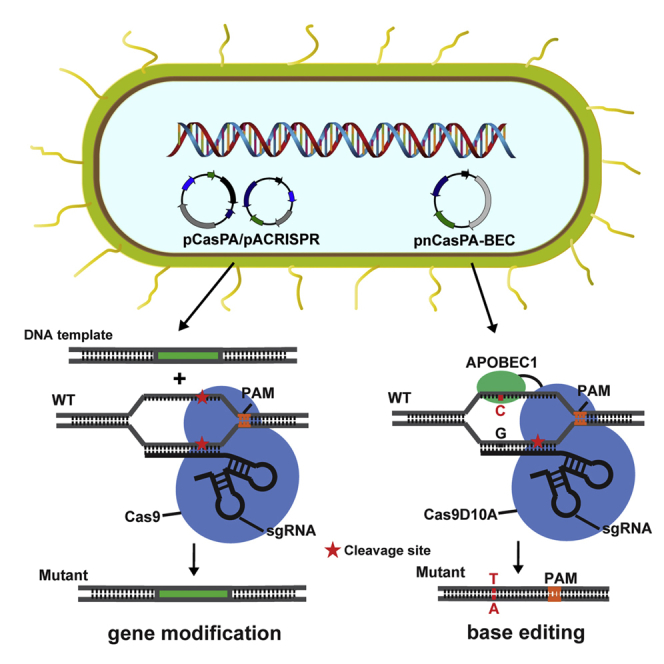

## Introduction

*Pseudomonas* is a genus of gram-negative Gammaproteobacteria existing in diverse environments, such as soil, water, plant, and human body (Palleroni, 1993, Peix et al., 2009). The significant biomedical, ecological, and industrial importance of these bacteria has rendered them as an excellent focus for scientific research (Gross and Loper, 2009, Peix et al., 2009). For instance, *Pseudomonas aeruginosa* is a major human pathogen and is capable of causing severe infections in vulnerable patients hospitalized with cystic fibrosis, burns, acquired immunodeficiency syndrome, or cancer. Extensive research has been focused to dissect the molecular basis of the infection mechanisms and to develop novel therapeutic means against *P*. *aeruginosa* infections (Hauser et al., 2011, Sadikot et al., 2012). *Pseudomonas putida* is a common soil bacterium and has been widely utilized for bioremediation and high-value chemical production (Moreno and Rojo, 2013, Nikel et al., 2016). Great efforts of metabolic engineering have been made to boost the capacity of *P*. *putida* in harsh environment and chemical production (Loeschcke and Thies, 2015, Wu et al., 2011).

Basic physiology studies and applied investigations in *Pseudomonas* species would benefit greatly from rapid and efficient genome editing tools. Despite the recent progress in the development of CRISPR-based genome engineering methods in *Pseudomonas* species, including the CRISPR-based systems for genome engineering in *P*. *putida* (Aparicio et al., 2017, Sun et al., 2018) and the dCas9-based transcription inhibition system in *Pseudomonas* (Tan et al., 2018), the genetic manipulation methods in most *Pseudomonas* species, such as the major human pathogen *P*. *aeruginosa*, are still time consuming and laborious (Hmelo et al., 2015, Hoang et al., 1998, Martínezgarcía and Lorenzo, 2011, Thomas et al., 2009). For instance, to construct a clean deletion mutant in *P*. *aeruginosa*, a two-step selection process is often required. First, a target gene is replaced by an antibiotic marker via homologous recombination. Second, the antibiotic marker is eliminated with the help of the FLP recombinase, leaving a scar sequence in place of the deleted gene (Hoang et al., 1998).

CRISPR/Cas9, obtained from bacterial adaptive immune systems (Horvath and Barrangou, 2010), has been engineered for genome editing in a variety of organisms, such as mammalian cells (Cong et al., 2013, Mali et al., 2013), *Saccharomyces cerevisiae* (Bao et al., 2015, Ryan and Cate, 2014), *Escherichia coli* (Jiang et al., 2013), and *Staphylococcus aureus* (Chen et al., 2017). In this system, the Cas9 DNA nuclease forms a complex with a single guide RNA (sgRNA). The complex can be precisely guided to any genomic locus via base pairing of the programmable 20-nucleotide (nt) sequence of sgRNA with the genomic DNA when an adjacent protospacer-adjacent motif (PAM, e.g., 5′-NGG-3′ for *Streptococcus pyogenes* Cas9 [Jinek et al., 2012]) is present in the target locus (Anders et al., 2014, Nishimasu et al., 2014, Wang et al., 2016, Wiedenheft et al., 2012). After binding, the Cas9 DNA nuclease cleaves the target locus, generating a double-stranded DNA break in the genome. Given that bacterial cells do not possess the non-homologous end-joining repair pathway, only the cells that have undergone homologous recombination can survive after the double-stranded DNA break of the genome. Thereby, it is possible to achieve a one-step seamless genome editing in *Pseudomonas* species with the utilization of the CRISPR/Cas9 system.

The recent development of “base editors” opens a new avenue for genome editing in biological systems. The systems directly catalyze the conversion of bases via a deamination reaction without generating a double-stranded DNA break or utilizing a donor repair template (Gaudelli et al., 2017, Komor et al., 2016). Until now, two kinds of base editors have been developed. The cytidine editor mediates the conversion of C→T (or G→A) (Banno et al., 2018, Gu et al., 2018, Komor et al., 2016), whereas the adenosine editor effects an A→G (or T→C) substitution (Gaudelli et al., 2017). The base editors are typically composed of a defective Cas9 protein (Cas9D10A or Cas9D10AH840A) and a deaminase fused to the Cas9 protein. Guided by the Cas9/sgRNA complex, the deaminase can be directed to any genomic locus to perform base editing in the single-stranded DNA (ssDNA) generated upon Cas9/sgRNA binding. By catalyzing the conversion of CAA, CAG, CGA, or TGG to TAA, TAG, or TGA codons, the cytidine base editor is capable of inactivating a target gene by generating a premature stop codon.

In this study, we developed a CRISPR/Cas9-mediated genome editing method, allowing for efficient and scarless genetic manipulation in *P*. *aeruginosa*. Moreover, we developed a base editing system, enabling highly efficient C→T (or G→A) conversion in *Pseudomonas* species. The two genome editing methods would greatly simplify the genetic manipulation in *Pseudomonas* species and accelerate a wide variety of investigations.

## Results

### Construction of CRISPR/Cas9-based Genome Editing System in *P*. *aeruginosa*

To develop an efficient and convenient genetic manipulation method in *Pseudomonas* species, we first sought to harness the CRISPR/Cas9 system for genome editing in these bacteria. We constructed a single-plasmid system pCasPAGm that possessed both the *Streptococcus pyogenes* Cas9 (spCas9) protein and the corresponding sgRNA expression cassettes (Figure S1A) (Jinek et al., 2012). We then assembled a 20-nt spacer and repair arms (∼1 kb each) into the plasmid to assess the editing efficiency of this system in a model *P*. *aeruginosa* strain PAO1. This system succeeded in deleting the *wspF* gene (GenBank: PA3703) with an efficiency of 9/11 (Figure S1B). However, it failed to delete the *nalD* (GenBank: PA3574) or *rhlR* (GenBank: PA3477) gene (Figures S1C and S1D). The discrepancy in the efficiency of the system in deleting different genes is likely ascribed to the weak intrinsic homologous recombination capacity of *P*. *aeruginosa*, which was further confirmed by the observation that only a small amount of colonies (10–20 colonies) could be obtained after editing even in the successful case of the deletion of the *wspF* gene.

**Figure 1:**
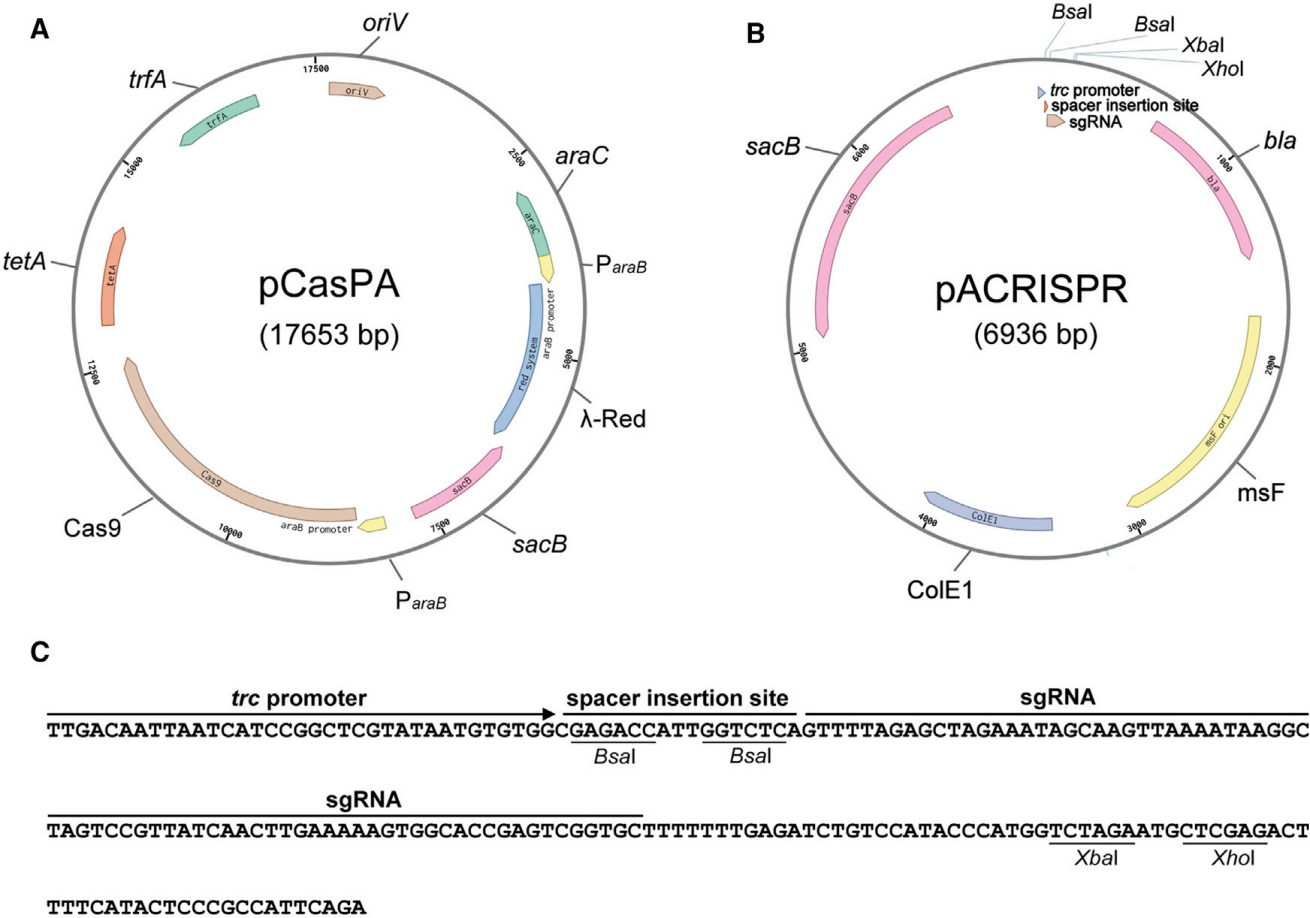
Maps and the Cloning Sites of the pCasPA/pACRISPR System (A) Map of the pCasPA plasmid. The expressions of both the Cas9 protein and the λ-Red system were driven by the L-arabinose-inducible *araB* promoter (P_*araB*_). The counter-selectable marker *sacB* was used for plasmid curing after editing. *tetA*, the tetracycline-resistance marker in *E*. *coli* and *P*. *aeruginosa*; *oriV*, the origin of replication; *trfA*, the essential gene for initiation of plasmid replication. (B) Map of the pACRISPR plasmid. *trc* Promoter, the sgRNA expression promoter; *bla*, the carbenicillin-resistance marker in *E*. *coli* and *P*. *aeruginosa*; mSF, a broad-host-range origin; ColE1, a replication origin for *E*. *coli*; *Bsa*I sites, Golden Gate assembly of spacers; *Xba*I and *Xho*I sites, Gibson assembly of repair arms; *sacB*, the counter-selectable marker for plasmid curing after editing. (C) Sequence of the cloning sites of the pACRISPR plasmid.

Phage recombination systems, such as λ-Red and RecET, have a greater homologous recombination capacity and have been engineered alone or in a couple of CRISPR systems for genetic manipulation in a variety of organisms (Datsenko and Wanner, 2000, Jiang et al., 2013, Jiang et al., 2015, Jiang et al., 2017, Murphy, 2012, Oh and van Pijkeren, 2014, Zhang et al., 1998). Thereby, we sought to increase the efficiency of homologous-recombination-mediated double-stranded DNA break repair in *P*. *aeruginosa* by introducing the λ-Red recombination system into the organism. Direct installation of the λ-Red system into the one-plasmid system pCasPAGm did not improve the efficiency of deleting the *nalD* gene (Figures S1E and S1F). Inspired by the successes of using a two-plasmid system for CRISPR/Cas9-mediated genome editing in *E*. *coli* and other bacteria (Jiang et al., 2013, Jiang et al., 2015), we designed and constructed a two-plasmid system pCasPA/pACRISPR. The pCasPA plasmid was capable of expressing the Cas9 nuclease and the λ-Red system proteins, Exo, Gam, and Bet. The expression of both the Cas9 nuclease and the λ-Red system was driven by the L-arabinose-inducible promoter P_*araB*_ (Narayanan et al., 2006) (Figure 1A). The pACRISPR plasmid was used to express the sgRNA. The expression of the sgRNA was driven by the well-studied strong promoter *trc* (Brosiuss et al., 1985) (Figure 1B). The pACRISPR plasmid contained two seamless cloning sites (Figure 1C). The *Bsa*I sites were used for the insertion of the 20-nt spacer by Golden Gate assembly (Engler et al., 2009), and the *Xba*I and *Xho*I sites were utilized for one-step cloning of repair arms by Gibson assembly (Gibson et al., 2009). The counter-selectable *sacB* that worked as a lethal gene in the presence of sucrose was introduced into both plasmids to facilitate rapid plasmid curing after editing (Schweizer, 1992).

### Assessment of the Editing Efficiency of the pCasPA/pACRISPR System

**Figure 2:**
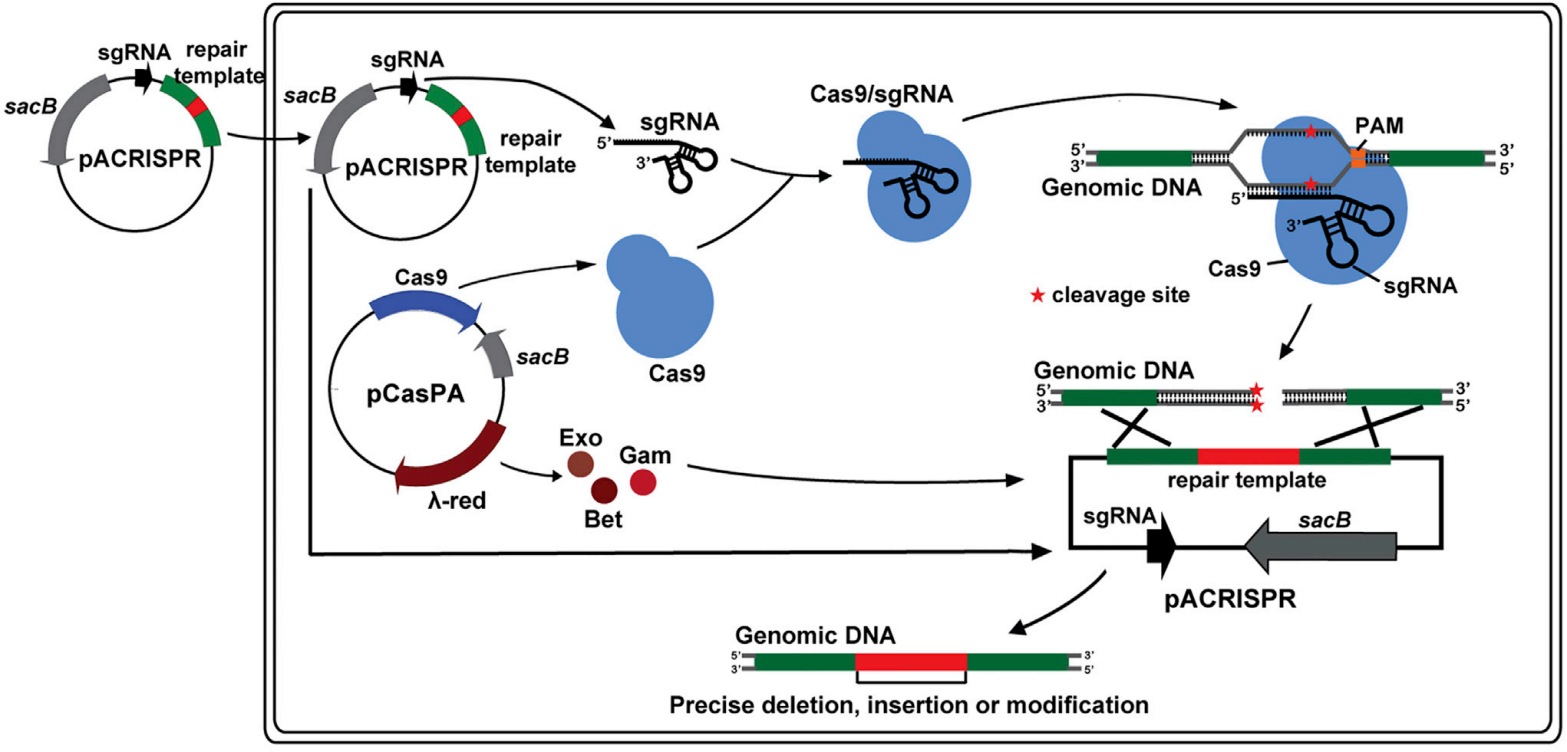
Scheme of pCasPA/pACRISPR-Mediated Genome Editing in *P*. *aeruginosa* The CRISPR/Cas9 system cleaves the target genome, generating a double-stranded DNA break. The λ-Red recombination proteins (Exo, Gam, and Bet) expressed by the pCasPA plasmid mediates the double-stranded DNA break repair by homologous recombination, resulting in precise genome modifications. The red asterisks are the cleavage sites of Cas9 protein.

**Figure 3:**
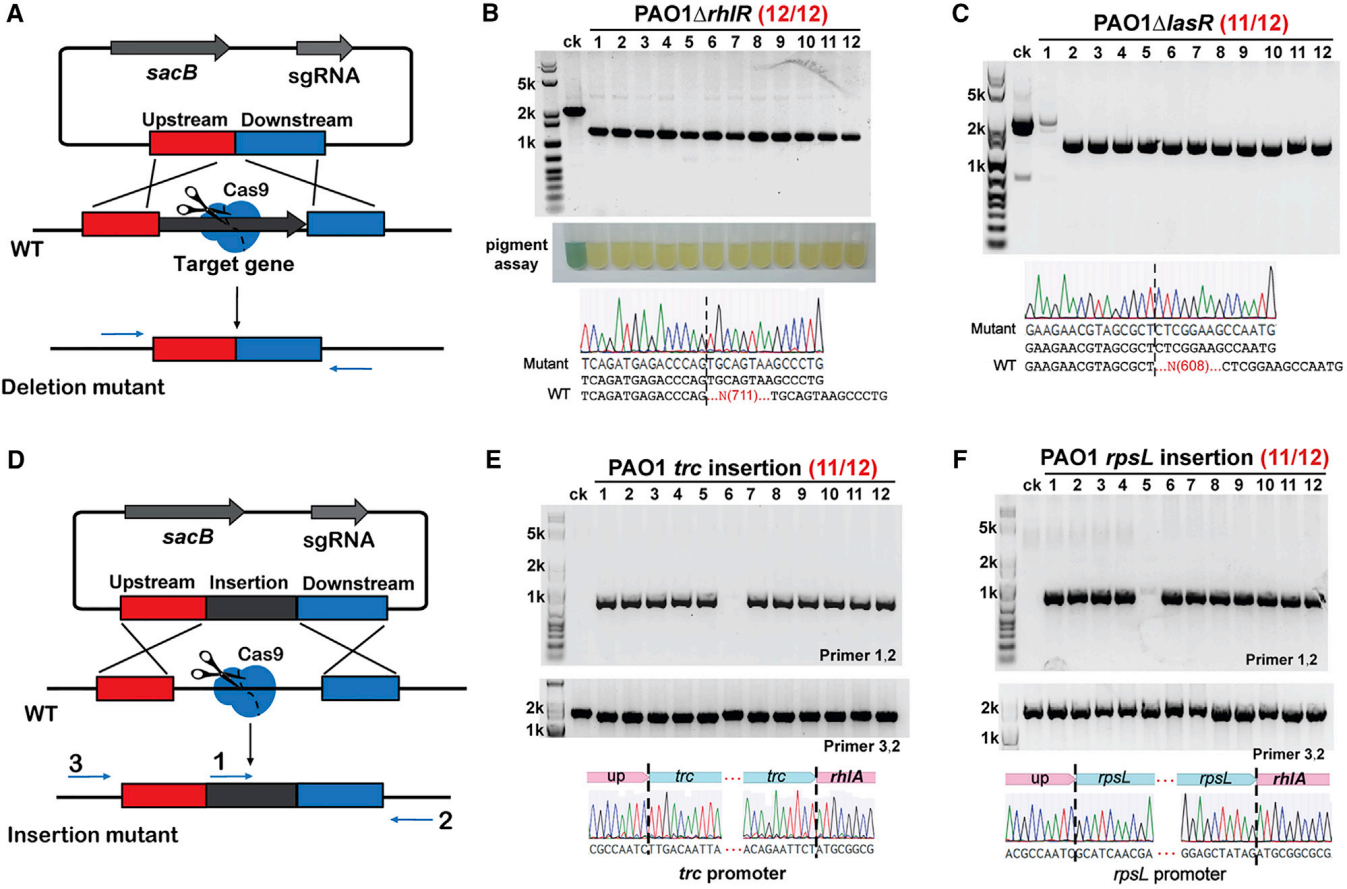
The pCasPA/pACRISPR System Enabled Highly Efficient Genome Editing in the *P*. *aeruginosa* PAO1 Strain (A) Schematic illustration of the procedures for gene deletion by the pCasPA/pACRISPR system. The blue arrows were the primers used for PCR validation of the editing efficiency. (B) The pCasPA/pACRISPR system allowed for highly efficient *rhlR* gene deletion in the PAO1 strain. The efficiency was 12/12, confirmed by PCR, sequencing, and pigment assay. (C) The pCasPA/pACRISPR system allowed for highly efficient *lasR* gene deletion in the PAO1 strain. The efficiency was 11/12, confirmed by PCR and sequencing. (D) Schematic illustration of the procedures for gene insertion. The blue arrows were the primers used for PCR validation. (E) The pCasPA/pACRISPR system enabled efficient *trc* promoter insertion in the PAO1 strain with an efficiency of 11/12. (F) The pCasPA/pACRISPR system enabled efficient *rpsL* promoter insertion in the PAO1 strain with an efficiency of 11/12.

To test the functionality of the pCasPA/pACRISPR system in genome editing in *P*. *aeruginosa*, we first transformed the pCasPA plasmid into the PAO1 strain. After the induction by l-arabinose for 2 hr, the cells containing the pCasPA plasmid were collected and prepared as the electrocompetent cells. Next, the empty pACRISPR plasmid, pACRISPR assembled with a 20-nt *rhlR* spacer (pACRISPR-*rhlR*_spacer), pACRISPR assembled with the *rhlR* repair arms (pACRISPR-*rhlR*_repair), as well as pACRISPR assembled with both spacer and repair arms (pACRISPR-*rhlR*) were electroporated into the cells for genome editing (Figures 2 and 3A). As shown in Figure S2A, more than 10^3^ colonies were observed for a single transformation with the pACRISPR and pACRISPR-*rhlR*_repair plasmid, whereas fewer than 10 colonies could be obtained for the same transformation with the pACRISPR-*rhlR*_spacer plasmid. The introduction of the *rhlR* spacer will produce an intact sgRNA that directs the Cas9 endonuclease to the *rhlR* gene locus to create a double-stranded break, leading to the death of cells. Around 100 colonies could be recovered when the cells were transformed with the pACRISPR-*rhlR* plasmid containing both the spacer and the repair arms. The *rhlR* gene in the colonies that was electroporated with the pACRISPR-*rhlR* plasmid was successfully deleted with an efficiency of 12/12, confirmed by PCR, sequencing, and the pigment production assay (loss of pigment production is a major phenotype for *rhlR* disruption [Brint and Ohman, 1995, Cao et al., 2014]) (Figure 3B). In addition, we also transformed the pACRISPR-*rhlR* plasmid into the pCasPA-containing PAO1 strain without the L-arabinose induction. As shown in Figure S2A, only several colonies were recovered on the plate, confirming that the λ-Red system is essential for the recombination and the leakage expression of the Cas9 protein is sufficient for the double-stranded DNA break.

Because the λ-Red recombination system is capable of promoting homologous recombination with the utilization of short (∼50 bp each) repair templates (Liang and Liu, 2010), we systematically investigated the editing efficiencies of the two-plasmid system when different lengths of repair templates were utilized. First, we tested the editing efficiencies of using the repair templates cloned into the pACRISPR plasmid. As shown in Figure S2B, the efficiencies of deleting the *nalD* gene were 100% when 500 bp or longer repair arms were utilized. However, the editing efficiency reduced significantly when shorter repair templates were utilized. No desired mutants could be obtained when a 45-bp repair arm was utilized (Figure S2B). In addition to the circular double-stranded DNA (cloned into the pACRISPR plasmid in this case), the λ-Red recombination system is also capable of using linear ssDNA or double-stranded DNA for recombination (Datsenko and Wanner, 2000, Ellis et al., 2001). Thereby, we tested the editing efficiency of the two-plasmid system when different lengths of linear ssDNA or double-stranded DNA were utilized as the repair templates. We co-transformed the linear repair templates and the pACRISPR plasmid into the cells that contained the pCasPA plasmid. The editing efficiencies of using the linear 45-nt single-strand DNA, 100-bp double-stranded DNA, 200-bp double-stranded DNA, 500-bp double-stranded DNA, and 1-kb double-stranded DNA were 2/12, 3/12, 9/12, 5/12, and 1/12, respectively (Figure S2C). The reduced editing efficiency of using 500-bp or longer linear repair templates is likely ascribed to the decreased transformation efficiency of long linear DNAs (Ghanta et al., 2018).

Given the differential editing efficiencies of using different types and lengths of the repair templates, we used 500-bp circular repair templates (assembled into the pACRISPR plasmid) for the subsequent genome editing experiments. We tested the efficiencies of the two-plasmid system in the deletions of four other genes in the PAO1 strain. The efficiencies of deleting *lasR* (GenBank: PA1430), *rsaL* (GenBank: PA1431), *algR* (GenBank: PA5261), and *rhlB* (GenBank: PA3478) genes were 11/12, 12/12, 5/12, and 8/12, respectively (Figures 3C and S3A).

To assess the capacity of the pCasPA/pACRISPR system in large-fragment deletion, we constructed three plasmids pACRISPR-*rhlR*3k, pACRISPR-*rhlR*5k, and pACRISPR-*rhlR*10k that were used to delete a 3-, 5-, and 10-kb DNA fragment, respectively, in the *rhlR* gene locus. As shown in Figure S3B, the 3-kb DNA fragment could be deleted with an efficiency of 12/12. However, the attempts to delete both the 5- and 10-kb DNA fragments failed. In addition to large-fragment deletion, we applied the pCasPA/pACRISPR system for multiplex gene editing. We cloned the spacers and repair arms of both the *rhlR* and *lasR* genes into a single pACRISPR plasmid and transformed it into the bacterial cells for editing. As shown in Figure S3C, the *rhlR* and *lasR* genes could be deleted simultaneously. However, the transformation colony-forming units (CFUs) decreased dramatically. Only 10–20 colonies were obtained from a single transformation (Figure S3D).

We noticed that some colonies could escape the CRISPR-induced death in the editing experiments. To probe the possible mechanism, we picked 4 escaped transformants from the PAO1Δ*nalD* plate. First, we sequenced the target gene (*nalD* gene locus) and no mutations were observed. Second, we tried to amplify the sgRNA fragment (∼1 kb) in the editing plasmid of the escaped transformants. As shown in Figure S3E, no band of ∼1 kb was observed for the 4 escaped transformants, whereas a clear band of ∼1 kb could be observed for the transformant that was successfully edited (ck). The results revealed that the editing plasmids from the escaped transformants contained mutations or deletions in DNA sequences coding for Cas9/sgRNA.

In addition to the genome editing in the *P*. *aeruginosa* PAO1 strain, we investigated the editing efficiency of the pCasPA/pACRISPR system in another widely utilized *P*. *aeruginosa* strain PAK. The efficiencies of deleting *rsaL*, *algR*, and *lasR* genes in the PAK strain were 10/12, 8/12, and 6/12, respectively (Figure S3F). In addition to gene deletion, we assessed the capacity of the two-plasmid system in gene insertion in *P*. *aeruginosa*. The native *rhlA* (GenBank: PA3479) promoter was successfully replaced by two foreign *trc* and *rpsL* promoters, both with efficiencies of 11/12 (Figures 3D and 3F). Together, these experiments demonstrated that the two-plasmid system pCasPA/pACRISPR possessed a great capacity for genome editing in *P*. *aeruginosa*.

### Plasmid Curing after Editing

To cure the plasmids after editing, one colony from the PAO1 strain containing the desired *nalD*-gene deletion was cultured in fresh Luria-Bertani (LB) medium until growth was evident. The culture was diluted for 10^4^ folds with fresh LB medium, and 100 μL diluted culture was plated onto the LB plates in the presence or absence of 5% w/v sucrose. Notably, much fewer colonies grew on the plate that had sucrose than on the plate that did not have sucrose (Figure S3G). Six individual colonies from the plate containing sucrose were randomly picked and streaked onto three different LB agar plates (no antibiotics, 100 μg/mL tetracycline, and 150 μg/mL carbenicillin). All the six colonies grew normally on the plate without antibiotics, whereas no growth of colonies was observed on the plates containing tetracycline or carbenicillin (Figure S3H), thus confirming that both the pCasPA and the pACRISPR plasmids could be easily cured after editing.

### Construction of the Base-Editing System pnCasPA-BEC

**Figure 4:**
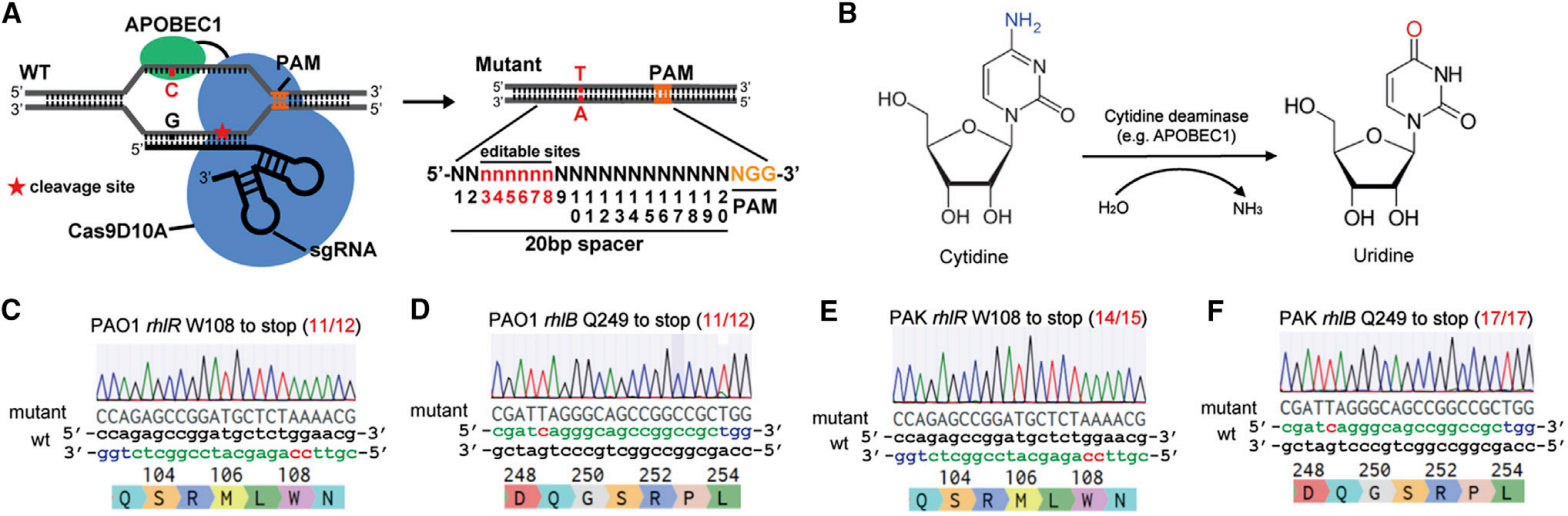
The Base Editor pnCasPA-BEC Enabled Highly Efficient C→T Conversion in *P*. *aeruginosa* (A) Schematic illustration of the “base editor”-mediated genome editing method. The potential editable sites of the pnCasPA-BEC system were highlighted in red. (B) The deamination reaction catalyzed by the cytidine deaminase. (C) The *rhlR* gene of the *P*. *aeruginosa* PAO1 strain was effectively inactivated by the pnCasPA-BEC system. The mutation efficiency of *rhlR* W108 to stop codon was 11/12. The mutation sites were colored red. See also Figure S4B. (D) The *rhlB* gene of the *P*. *aeruginosa* PAO1 strain was effectively inactivated by the pnCasPA-BEC system. The mutation efficiency of *rhlB* Q249 to stop codon was 11/12. (E) W108 of the *rhlR* gene in the *P*. *aeruginosa* PAK strain was successfully mutated to stop codons with an efficiency of 14/15. (F) Q249 of the *rhlB* gene in the *P*. *aeruginosa* PAK strain was successfully mutated to stop codons with an efficiency of 17/17.

To expand the utility of the pCasPA/pACRISPR system, we assessed the capacity of this system for genome editing in other widely studied microbes, including *P*. *putida*, *Pseudomonas fluorescens* (a plant growth-promoting bacterium), and *Pseudomonas syringae* (a major plant pathogen). However, the extremely low efficiency for the transformation of the pCasPA plasmid into these microbes prevents its applications in genome editing in these bacteria. The large size of the pCasPA plasmid (17653 bp) and the toxicity of the Cas9 protein and the λ-Red system may be the possible reasons for the low transformation efficiency (Jiang et al., 2014, Sun et al., 2018). Thereby, we sought to develop a base editing system as an alternative way for genome editing in these bacteria, because base editing systems have been demonstrated to be emerging tools for genetic manipulation in microbes without generating double-stranded DNA break or sacrificing transformation CFUs (Gu et al., 2018). We designed and constructed a base editing system pnCasPA-BEC (Figure S4A). In this plasmid, the cytidine deaminase (rat APOBEC1) was fused to the N terminus of the Cas9 nickase (SpCas9D10A) via an XTEN linker (Komor et al., 2016) (Figures 4A and 4B). The expression of the fusion protein and the sgRNA were driven by the *rpsL* promoter (from PAO1) and the *trc* promoter, respectively. Two *Bsa*I sites were engineered into the plasmid for seamless cloning of the 20-bp spacer using Golden Gate assembly. The broad-host-range replicon mSF was introduced in this system for plasmid replication in *Pseudomonas* species. In addition, we utilized the *sacB* gene for plasmid curing after editing.

### Assessment of the Editing Efficiency of the pnCasPA-BEC System in *Pseudomonas* Species

We first assessed the capacity of the pnCasPA-BEC system in base editing in the PAO1 strain. We designed spacers of *rhlR* and *rhlB* genes containing potential editable C(s) within the editable window, which was reported from positions 4 to 8 in mammalian cells (Komor et al., 2016). The spacers were assembled into the pnCasPA-BEC plasmid, and the constructed plasmids were transformed into the PAO1 strain. In agreement with our expectation, the Cs at positions 6 and 7 of the *rhlR* spacer, and the C at position 5 of the *rhlB* spacer, were successfully mutated to T with an efficiency of 11/12 for both two genes (Figures 4C, 4D, and S4B). These conversions generated premature stop codons in the genes, resulting in gene inactivation. In addition, this system succeeded in base editing in the *P*. *aeruginosa* PAK strain with high efficiencies (14/15 for *rhlR* and 17/17 for *rhlB*) (Figures 4E and 4F). After editing, the plasmid could be easily cured by plating the cells on the plate containing sucrose (Figure S4C).

To assess the nonspecific mutator effects of pnCasPA-BEC, we searched spacers similar to the *rhlR* and *rhlB* spacers across the entire genome using sgRNAcas9 software (Xie et al., 2014). We picked the top six similar spacers for each gene (Table S1). Next, we amplified the spacer locus and sent them out for sequencing. The results showed that none of the similar spacer sites were mutated (Table S1).

**Figure 5:**
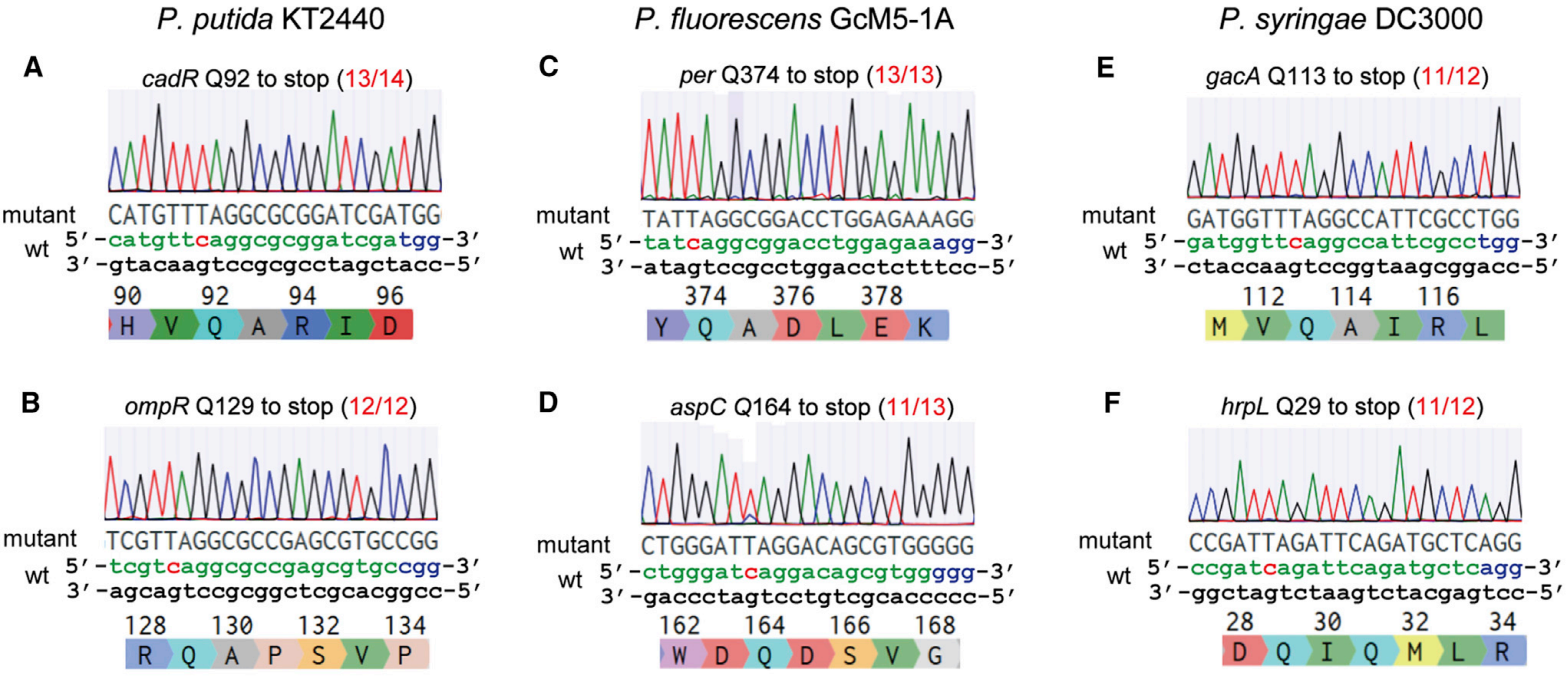
The pnCasPA-BEC System Enabled Highly Efficient C→T Conversion in a Variety of *Pseudomonas* Species (A and B) *P*. *putida* KT2440 *cadR* Q92 (A) and *ompR* Q129 (B) were successfully mutated to stop codons with efficiencies of 13/14 and 12/12, respectively. (C and D) *P*. *fluorescens* GcM5-1A *per* Q374 (C) and *aspC* Q164 (D) were mutated to stop codons with efficiencies of 13/13 and 11/13, respectively. (E and F) *P*. *syringae* DC3000 *gacA* Q113 (E) and *hrpL* Q29 (F) were mutated to stop codons with efficiencies of 11/12 and 11/12, respectively.

Next, we applied the pnCasPA-BEC system for base editing in other *Pseudomonas* species, including *P*. *putida*, *P*. *fluorescens*, and *P*. *syringae*. As shown in Figures 5A–5F, all the *cadR* (GenBank: PP_5140) and *ompR* (GenBank: PP_0246) genes in *P*. *putida* KT2440, the *per* (GenBank: GU120326) and *aspC* (GenBank: FJ485937) genes in *P*. *fluorescens* GcM5-1A, as well as the *gacA* (GenBank: PSPTO_3024) and *hrpL* (GenBank: PSPTO_1404) genes in *P*. *syringae* DC3000 were successfully mutated with high efficiencies, demonstrating the great capacity of the pnCasPA-BEC system for base editing in a variety of *Pseudomonas* species.

To systematically investigate the editable window of the pnCasPA-BEC system in *Pseudomonas* species, we designed and assembled eight different spacers containing Cs at positions from 2 to 9 into the pnCasPA-BEC plasmid. Next, we transformed the plasmids into the PAO1 strain and examined the editing efficiencies. The results showed that all the Cs at positions 3 to 8 were mutated to Ts with an efficiency of 100%, whereas the Cs at the position 2 or 9 could not be mutated (Figure S4D). Thereby the possible editable window of the pnCasPA-BEC system was from positions 3 to 8 in the PAO1 strain (Figure S4D). Notably, the adjacent bases of the editable sites greatly affected the editing efficiency. The editing efficiencies of GC and AC were much lower than those of CC and TC (Figures 4C, 4D, S4D, and S4E), in agreement with the results of mammalian cells (Komor et al., 2016).

## Discussion

We have engineered the CRISPR/Cas9 and the λ-Red recombination systems (pCasPA/pACRISPR) for rapid, precise, and seamless genetic manipulation in *P*. *aeruginosa*. We first directly installed the λ-Red system and CRISPR/Cas9 into a single plasmid, but the resulting plasmid failed to improve the editing efficiency (Figures S1E and S1F). From our experiences and others' work (Jiang et al., 2013, Sun et al., 2018), to achieve a successful editing, the λ-Red recombination proteins probably need to be pre-expressed in bacterial cells before the genome cleavage by CRISPR/Cas9. Thus, when both the CRISPR/Cas9 system and the λ-Red system are present in a single plasmid, the λ-Red system may be expressed too late to repair the double-stranded DNA break generated by the CRISPR/Cas9 system that is expressed at the same time as the λ-Red system in the one-plasmid system. These observations may shed light on the development of CRISPR/Cas9-based genome editing tools in other bacteria.

We systematically investigated the optimal editing conditions of the pCasPA/pACRISPR system. Both circular and linear templates could be used for homology-directed repair (Figures S2B and S2C). Although short linear ssDNA repair template (e.g., 90 bp) could be synthesized easily and used for repair directly, its repair efficiency was relatively low (Figure S2C). Long circular template (>500 bp) exhibited high repair efficiency (Figure S2B). However, it had to be cloned into the editing plasmid for repair. The pCasPA/pACRISPR system also enabled multiplex gene editing (Figure S3C). However, the complex construction process and low-transformation CFUs may limit its application.

The highly efficient base-editing system pnCasPA-BEC is capable of inactivating genes in a variety of *Pseudomonas* species. Compared with the recently reported Cpf1-BEC that recognizes AT-rich (TTTV) PAM (Li et al., 2018), the Cas9-BEC that recognizes the NGG PAM is more suitable for base editing in *P*. *aeruginosa*, because the genome of *P*. *aeruginosa* is GC rich. No off-target mutations were detected in the six similar spacers of the *rhlR* and *rhlB* genes. The editing window of the pnCasPA-BEC system is from positions 3 to 8 in the PAO1 strain, which is slightly different from that of the base editors in the mammalian cells (Komor et al., 2016) and *S*. *aureus* (Gu et al., 2018). Given the ease of use and high efficiency, future engineering of the base-editing system would provide a new way for high-throughput screening in *Pseudomonas* species.

## Methods

All methods can be found in the accompanying Transparent Methods supplemental file. The strains, plasmids, and primers used in this study are listed in Tables S2, S3, and S4, respectively.

## Supplemental Information

Document S1. Transparent Methods, Figures S1–S4, and Tables S1–S3

Table S4. Primers Used in this Study, Related to Figures 1 and 3–5 and Transparent Methods
