# Supplementary material for: CRISPR/Cas9-based Genome Editing in Pseudomonas aeruginosa and Cytidine Deaminase-Mediated Base Editing in Pseudomonas Species
Source: iScience. 2018 Aug 1;6:222–31. doi: 10.1016/j.isci.2018.07.024 (PMC6137401; doi:10.1016/j.isci.2018.07.024)
Supplement: Document S1. Transparent Methods, Figures S1–S4, and Tables S1–S3 [file mmc1.pdf]

ISCI, Volume *6*

Supplemental Information

CRISPR/Cas9-based Genome Editing in *Pseudomonas aeruginosa* and Cytidine Deaminase-Mediated Base Editing in *Pseudomonas* Species

Weizhong Chen, Ya Zhang, Yifei Zhang, Yishuang Pi, Tongnian Gu, Liqiang Song, Yu Wang, and Quanjiang Ji

Transparent Methods

Bacterial Strains, Plasmids, Primers and Growth Conditions

The strains, plasmids, and primers used in this study were listed in tables S2, S3 and S4, respectively. All *E. coli* strains and *Pseudomonas* strains were grown in Luria-Bertani broth (LB). *E. coli* strains and *P. aeruginosa* strains were grown at 37 ^{o}C, while *P. putida*, *P. fluorescens*, and *P. syringae* were cultured at 30 ^{o}C.

Plasmid Construction

The construction of the pCasPA plasmid was performed using the following method. The λ-Red system and the *araB* promoter (P_{araB}) were amplified from the pKD46 plasmid, respectively (Datsenko and Wanner, 2000). The gene encoding the Cas9 nuclease was amplified from the pCasSA plasmid (Chen et al., 2017). These three DNA fragments were assembled between the *Kpn*I/*Hind*III sites of the pDN19 plasmid (Li et al., 2013) using Gibson assembly. Next, the *sacB* gene amplified from the pEX18Ap plasmid (Hoang et al., 1998) was inserted into the *Xba*I/*Xho*I sites of the aforementioned plasmid, resulting in the final plasmid pCasPA. The pACRISPR plasmid was constructed by inserting the *trc* promoter along with the sgRNA and the *sacB* gene into the *BamH*I/*Hind*III sites of the pAK1900 vector(Srikumar et al., 1998) via Gibson assembly.

The pnCas9PA-BEC plasmid was constructed using the following procedures. The *rpsL* promoter was amplified from the genomic DNA of the *P. aeruginosa* PAO1 strain. The origin ColE1 and the gene encoding APOBEC1-Cas9D10A were amplified from the pnCasSA-BEC plasmid (Gu et al., 2018). The *sacB* gene was amplified from the pEX18Ap plasmid (Hoang et al., 1998). The origin mSF was amplified from pAK1900 plasmid (Srikumar et al., 1998). The gentamycin-resistance marker was amplified from the pPS858 plasmid (Hoang et al., 1998). The six fragments were assembled into a plasmid via Golden Gate assembly. The *trc* promoter along with the sgRNA was amplified from the pACRISPR plasmid and inserted into the *Sal*I/*Xho*I sites of the aforementioned plasmid, resulting in the final pnCasPA-BEC plasmid.

Preparation of Electrocompetent Cells

1 mL overnight culture of *P. aeruginosa* was 1:100 diluted into 100 mL of fresh LB medium and incubated at 37 ^{o}C. When the optical density at 600 nm (OD_{600}) of the culture reached ~1.0, the cells were harvested by centrifugation at 6000 rpm for 5 min. The supernatant was discarded, and the cells were washed twice with 20 mL of sterile ice-cold 10% v/v glycerol. Finally, the cells were resuspended into 1 mL of 10% v/v glycerol. 50 μL aliquots of the cells were used for the subsequent experiments. Similar procedures as that of the preparation of electrocompetent *P. aeruginosa* cells were utilized to make *P. putida*, *P. fluorescens*, and *P. syringae* electrocompetent cells.

Genome Editing Using the pCasPA/pACRISPR System

The pCasPA plasmid was transformed into *P. aeruginosa* strains using electroporation with the parameters of 2100 V, 100 Ω, 25 μF, 1 mm cuvette (Bio-Rad). The colonies containing pCasPA were selected on a LB agar plate in the presence of 100 μg/mL tetracycline. A colony was picked from the plate and cultured in the LB medium at 37 ^{o}C overnight. The next day, 1 mL culture was diluted into 100 mL fresh LB medium and shaken at 37 ^{o}C until the OD_{600} of 1.0~1.5. The expression of the Cas9 nuclease and the λ-Red system was induced by the addition of L-arabinose to a final concentration of 2 mg/mL. The culture was further incubated for 2 hours before being prepared as the electrocompetent cells. Next, the pACRISPR plasmid assembled with the spacer and the repair template was electroporated into the electrocompetent cells. After electroporation, the cells were recovered in LB for 1~2 hours at 37 ^{o}C and plated onto the LB agar plate containing 100 μg/mL tetracycline and 150 μg/mL carbenicillin. PCR and sequencing were utilized to evaluate the editing efficiency. The editing efficiency was calculated as the number of colonies mutated successfully divided by the number of all the colonies evaluated.

Base Editing Using the pnCasPA-BEC Plasmid

For the base editing in the *P. aeruginosa* strains, a suitable 20-bp spacer sequence before a PAM site in the target locus was designed and inserted into the *BsaI* sites of the pnCasPA-BEC plasmid using Golden Gate assembly. The constructed plasmid was then transferred into *P. aeruginosa* strains and the colonies were selected on the LB agar plate containing 30 μg/mL gentamycin. The PCR reaction was performed individually using the genomic DNA of colonies as the template, and the PCR products covering the editable site(s) were sent out for sequencing. The base editing of other *Pseudomonas* spp. such as *P. putida*, *P. fluorescens*, and *P. syringae* were performed following a similar procedure, except that these three strains were selected on the LB plate containing 20 μg/mL gentamycin.

Pigment Assay

Different colonies were randomly picked and cultured in 3 mL pyocyanin production broth (PPB) medium (PPB medium: 20 g peptone, 1.4 g $MgCl_2$, 10 g K_2SO_4, 20 mL glycerol per liter; pH 7.0)(Brint and Ohman, 1995). One colony of the PAO1 wild-type strain was incubated in the same condition as a control. The tubes were shaken at 37 ^{o}C for 12~16 h before being photographed.

Data and Software Availability

The plasmids have been deposited in the Addgene (http://www.addgene.org/) with the accession codes 113347 (pCasPA), 113348 (pACRISPR) and 113348 (pnCasPA-BEC).

Detailed protocol for the construction of the pACRISPR plasmid.

(1) Spacer design

Design a suitable 20 nt DNA sequence in the target gene of *P. aeruginosa* via sgRNAcas9(Xie et al., 2014). Two oligos were designed in the following form:

5′-**GTGG**NNNNNNNNNNNNNNNNNNNN-3′

3′-NNNNNNNNNNNNNNNNNNNN**CAAA**-5′

(2) Phosphorylation of the oligos

Component	Volume
10× T4 DNA ligase buffer (NEB)	5 μL
50 μM Oligo F	2 μL
50 μM Oligo R	2 μL
T4 polynucleotide kinase (Takara)	1 μL
ddH_2O	40 μL
Total volume	50 μL

Incubate at 37 °C for 1 hour.

(3) Annealing

2.5 μL 1 M NaCl was added into the phosphorylated product. The phosphorylated oligos were incubated at 95 °C for 3 min and then slowly cooled down to room temperature using a thermocycler. Dilute the annealed oligos 20 folds to the final concentration of 100 nM with ddH_2O.

(4) Golden Gate assembly

Prepare the samples as indicated below:

Component	Volume
10× T4 DNA ligase buffer (NEB)	1 μL
Annealed oligos (100 nM)	1 μL
The pACRISPR plasmid (20 nM)	1 μL
T4 DNA ligase (NEB)	0.5 μL
*Bsa*I-HF (NEB)	0.5 μL
ddH_2O	6 μL
Total volume	10 μL

Run the reaction protocol in a thermocycler:

Segment	Temperature	Time	Cycles
1	37 ℃	3 min	25
	16 ℃	4 min	
2	80 ℃	15 min	1
3	10 ℃	Forever	1

(5) Transformation

The Golden Gate assembly product was transformed into 100 μL *E.coli* DH5α competent cells. The cells were plated onto a LB agar plate containing 50 μg/mL carbenicillin and incubated at 37 ℃ overnight. The success for the construction of the pACRISPR-NN_spacer plasmid was

verified by PCR and sequencing.

(6) Digest the pACRISPR-NN_spacer plasmid with *Xba*I and *Xho*I

Component	Volume
10× CutSmart buffer (NEB)	5 μL
2 μg pACRISPR-NN_spacer plasmid	xx μL
*Xba*I (NEB)	2 μL
*Xho*I (NEB)	2 μL
ddH_2O	xx μL
Total volume	50 μL

Incubate the mixture at 37 ℃ for 2~3 hours. The digested plasmid was purified by using the SanPrep PCR purification kit (Sangon Biotech, Shanghai, China).

(7) Gibson assembly

Select ~500bp DNA sequence of the upstream and downstream of the target gene, respectively. The two DNA fragments were PCR-amplified from the genomic DNA of the *p. aeruginosa* strains with a 20~40 bp overlap of the digested pACRISPR-NN_spacer plasmid. The overlap could be created via PCR with primers that contain a 5′ end that is identical to an adjacent segment and a 3′ end that anneals to the target sequence.

5' primer of the upstream is in this form:

5'- ttttgagatctgtccatacccatggTCTAGANNNNNNNNNNNNNNNNNNNNNNN -3'

3' primer of the downstream is in this form:

5'-tctgaatggcgggagtatgaaaagtCTCGAGNNNNNNNNNNNNNNNNNNNNNNN -3'

3' primer of the upstream and 5' primer of the downstream should have 30~40 bp overlap between each other.

The two DNA fragments were assembled into the digested pACRISPR-NN_spacer plasmid using Gibson assembly.

Component	Volume
NEBuilder HiFi DNA Assembly Master Mix (NEB)	10 μL
20 fmol *Xba*I/*Xho*I digested pACRISPR-NN_spacer plasmid	xx μL
20 fmol upstream of the target gene	xx μL
20 fmol downstream of the target gene	xx μL
ddH_2O	xx μL
Total volume	20 μL

The reaction solution was incubated at 50 ℃ for 1 hour.

For the gene insertion, external 20 fmol DNA fragment of the *trc* promoter or *rpsL* promoter was added into the reaction solution.

The Gibson assembly product was transformed into 100 μL *E.coli* DH5α competent cells. The cells were plated onto a LB agar plate containing 50 μg/mL carbenicillin. The successful construction of the plasmid pACRISPR-NN was verified by PCR and sequencing.

The construction of pnCasPA-BEC_spacer followed the same protocol of steps 1~4. The plasmid was selected on the LB agar plate containing 15 μg/mL gentamycin.

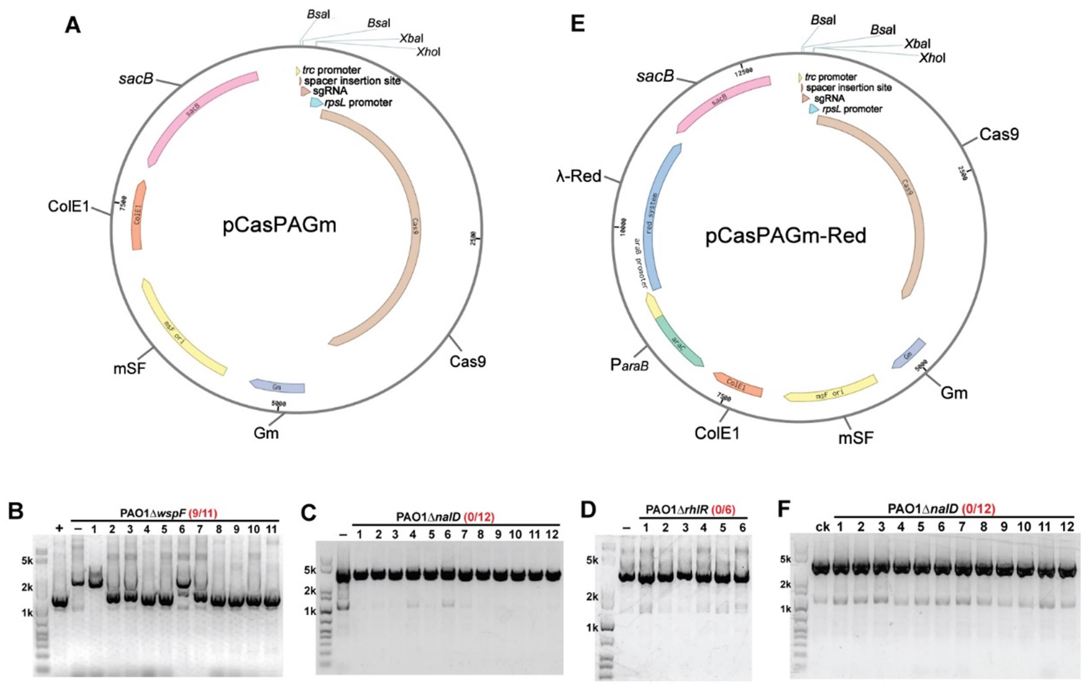

Figure S1. The single plasmid system pCasPAGm and pCasPAGm-Red deleted genes in *P. aeruginosa* with different efficiencies, related to Figure 1. (A) Map of the one-plasmid genome editing plasmid pCasPAGm. *trc* promoter, the sgRNA expression promoter; *rpsL* promoter, the Cas9 protein expression promoter; *Bsa*I sites, Golden Gate assembly of spacers; *Xba*I and *Xho*I sites, Gibson assembly of repair arms; mSF, A broad-host-range origin amplified from pAK1900 vector; ColE1, a replication origin for *E. coli*; Gm, the gentamycin-resistance marker in *E. coli* and *P. aeruginosa*; *sacB*, the counter-selectable marker for fast curing of the plasmid after editing. (B) The pCasPAGm system enabled the deletion of *wspF* gene in the PAO1 strain with the efficiency of 9/11. (C) The pCasPAGm system failed to delete *nalD* gene in the PAO1 strain. (D) The pCasPAGm system failed to delete *rhlR* gene in the PAO1 strain. (E) Map of the pCasPAGm-Red plasmid. The plasmid was constructed by the insertion of the λ-Red system into the pCasPAGm plasmid. (F) The pCasPAGm-Red system failed to delete the *nalD* gene in the PAO1 strain.

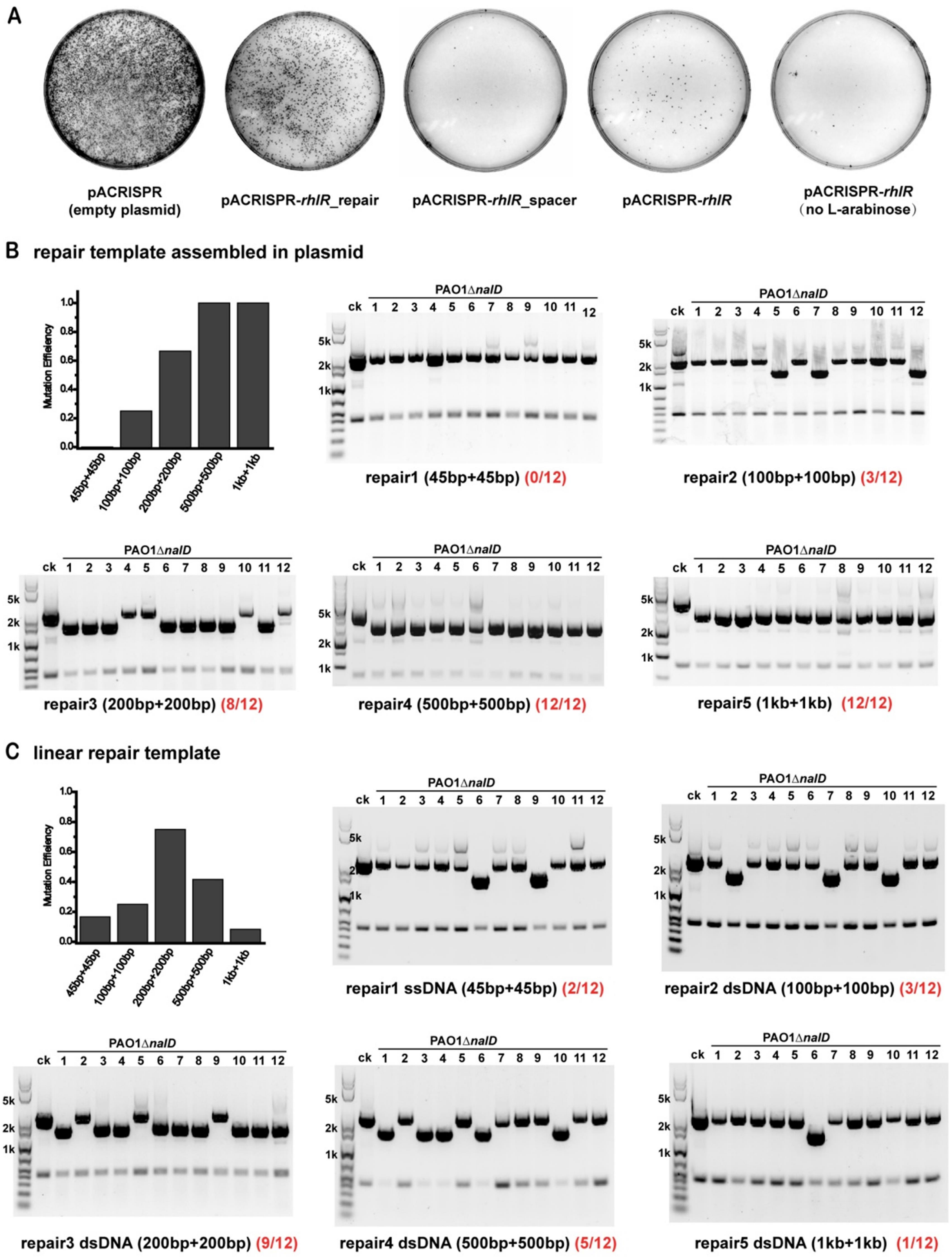

Figure S2. The pCasPA/pACRISPR system-mediated genome editing in the *P. aeruginosa* PAO1, related to Figure 3. (A) 1 µg pACRISPR (empty plasmid), pACRISPR-*rhlR*_spacer (assembled with a 20-nt *rhlR* gene spacer), pACRISPR-*rhlR*_repair (assembled with the *rhlR* gene repair arms), and pACRISPR-*rhlR* (assembled with both spacer and repair arms) plasmid was electroporated into the PAO1 strain containing pCasPA after L-arabinose induction, respectively. The pACRISPR-*rhlR* plasmid was also transformed into the pCasPA-containing PAO1 strain without arabinose induction as a control. (B) The

editing efficiency varies when different lengths of circular repair templates (assembled into the pACRISPR plasmid) were utilized for *nalD* gene deletion in the PAO1 strain. (C) The editing efficiency varies when different lengths of linear repair templates were utilized for *nalD* gene deletion in the PAO1 strain.

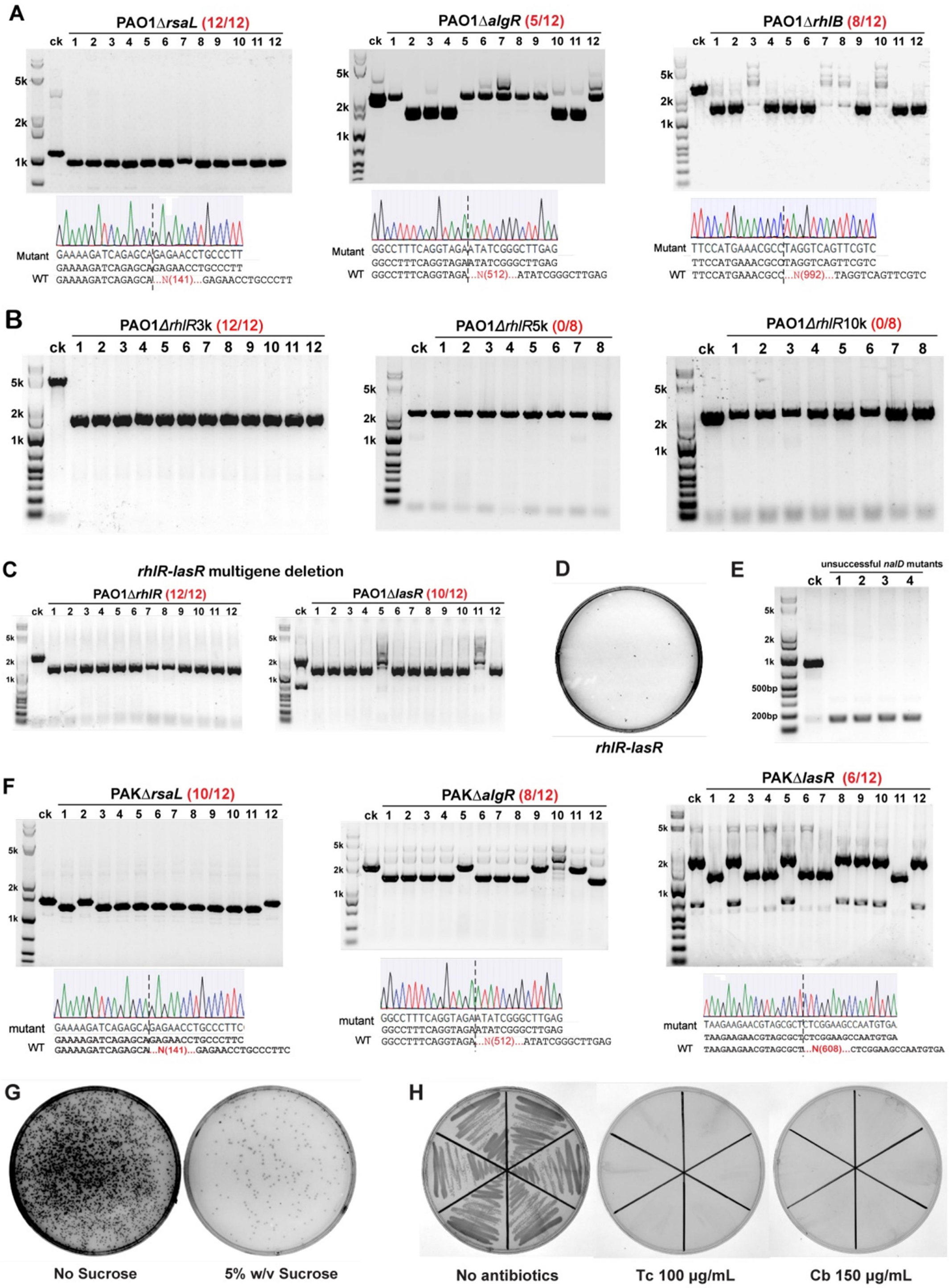

Figure S3. The pCasPA/pACRISPR system enabled highly efficient Genome editing in the *P. aeruginosa* PAO1 and PAK strains, and can be easily cured after editing, related to Figure 3. (A) pCasPA/pACRISPR-mediated deletion of the *rsaL*, *algR* and *rhlB* genes in the PAO1 strain with the efficiencies of 12/12, 5/12 and 8/12, respectively. The "ck" lane was the PCR product from the wild-type strain as a control. (B) The pCasPA/pACRISPR system successfully deleted 3kb DNA fragment in the *rhlR* gene locus with the efficiency of 12/12, while it failed in the deletion of 5kb and 10kb DNA fragments in the same locus. (C) The pCasPA/pACRISPR system enabled

multiplexed gene deletion. The *rhlR* and *lasR* genes were deleted simultaneously in a single transformation with the efficiency of 10/12. (D) The transformation CFUs of multiplexed gene deletion. Only 10~20 colonies were observed on the LB plate after electroporation with 1 μg of the pACRISPR-*rhlR-lasR* plasmid. (E) The sgRNA locus of the four escaped transformants from the PAO1*ΔnalD* plate could not be amplified. The "ck" lane was the PCR product from the mutant that was successfully edited as a control. (F) pCasPA/pACRISPR-mediated deletion of the *rsaL*, *algR* and *lasR* genes in the PAK strain with the efficiencies of 10/12, 8/12 and 6/12, respectively. (G) Much fewer colonies grew on the plate in the presence of sucrose (right) than that grew on the plate without sucrose (left). (H) The colonies picked from the plate containing sucrose failed to grow on the plates containing 100 μg/mL tetracycline (middle) or 150 μg/mL carbenicillin (right). Colonies only grew on the plate without antibiotics, indicating both the pCasPA and the pACRISPR plasmids were successfully cured.

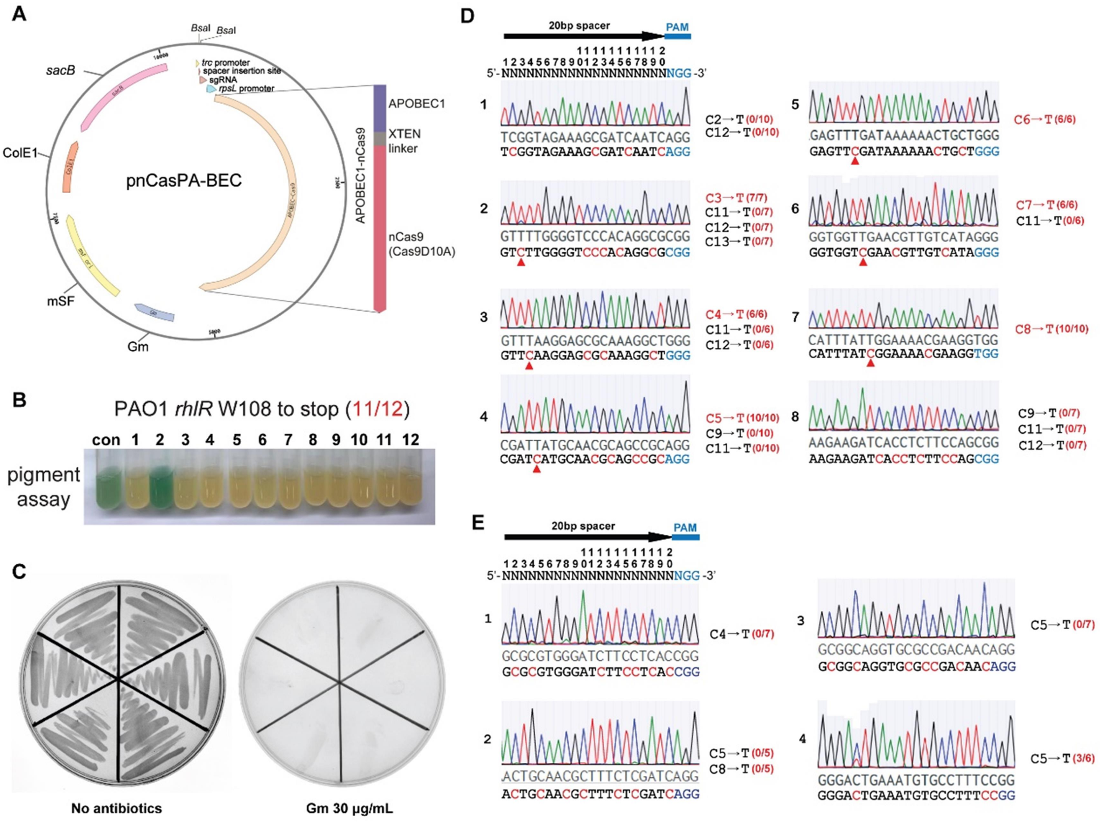

Figure S4. The C→T base editing plasmid pnCasPA-BEC in *P. aeruginosa*, related to Figure 4. (A) Map of the C→T base editing plasmid pnCasPA-BEC. APOBEC1-nCas9, a fusion protein composed of a deaminase APOBEC1 at the N terminus and a Cas9 nikase Cas9D10A at the C terminus; *trc* promoter, a strong promoter to drive the expression of the sgRNA; *rpsL* promoter, the APOBEC1-nCas9 protein expression promoter; *Bsa*I sites, Golden Gate assembly of spacers; mSF, A broad-host-range origin from pAK1900 vector; ColE1, a replication origin for *E. coli*; Gm, the gentamycin-resistance marker in *E. coli* and *P. aeruginosa*; *sacB*, the counter-selectable marker for fast curing of the plasmid after editing. (B) The *rhlR* gene of the PAO1 strain was effectively inactivated by the pnCasPA-BEC system. The mutation efficiency of *rhlR* W108 to stop codon was 11/12 confirmed by pigment assay. (C) The pnCasPA-BEC plasmid could be easily cured after editing. Six individual colonies picked from the plate containing sucrose were streaked onto the LB agar plates in the presence (right) or absence (left) of gentamycin. Colonies only grew on the plate without gentamycin, indicating the pnCasPA-BEC plasmid was successfully cured. (D) Examination of the editable window of the pnCasPA-BEC system. A representative sequencing chromatogram for the target locus was shown. The C(s) in the spacer were colored red, and the editable C(s) were indicated with red triangles. (E) The kind of adjacent bases of editable sites affected the editing efficiency greatly.

Table S1. The sequence and position of the spacer of *rhlR* and *rhlB* genes and their top six similar spacer sites, related to Figure 4.

Gene	Site	Sequence	Position	Mutation efficiency
rhlB	WT	cgatcagggcagccggccgctgg	3891315-3891293	11/12
	M1	c**C**atc**T**gggc**G**gccggccgctgg	841017-841039	0/4
	M2	cgatc**C**g**C**gcagcc**T**gccgctgg	3984809-3984831	0/4
	M3	cgatcaggg**TC**gccg**A**c**A**gctgg	1565062-1565040	0/4
	M4	cgatca**C**ggcagccg**C**c**AC**ctgg	4873175-4873197	0/4
	M5	cgatc**C**g**C**gcagcc**T**gccg**GC**gg	5005635-5005613	0/4
	M6	**T**gatcagg**T**c**G**gccggc**T**gc**A**gg	5534902-5534924	0/4
rhlR	WT	cgttccagagcatccggctctgg	3890323-3890345	11/12
	M1	cgttccaga**C**catcc**A**gc**G**c**GA**g	743768-743790	0/4
	M2	cg**C**tc**TC**gagcatccggc**G**ctgg	1905399-1905421	0/4
	M3	cg**GC**cc**T**gagca**C**ccggctc**G**gg	5090604-5090626	0/4
	M4	cgt**CGT**ag**T**gcatccggc**A**ctgg	591063-591085	0/4
	M5	cgttc**G**aga**T**c**C**tc**G**ggct**G**tgg	2514335-2514357	0/4
	M6	cgttc**TG**g**C**gca**G**ccggc**G**ctgg	2737948-2737926	0/4

The mismatched bases are labeled with the thicker capital letter.

Table S2. Bacterial strains used in this study. Related to Fig. 1, Fig. 2, Fig. 3, Fig.4, Fig. 5, and Transparent Methods.

strains	Description	Reference
E.coli		
DH5α	F^- Φ80*lac*ZΔM15 Δ(*lac*ZYA-*arg*F) U169 *rec*A1 *end*A1 *hsd*R17 (r_K^-, m_K^+) *pho*A *sup*E44 λ^- *thi*-1 *gyr*A96 *rel*A1	Lab stock
P. aeruginosa strain		
PAO1	Wild-type	Lab stock
PAO1Δ*rhlR*	PAO1 *rhlR* gene deleted	This study
PAO1Δ*nalD*	PAO1 *nalD* gene deleted	This study
PAO1Δ*rsaL*	PAO1 *rsaL*gene deleted	This study
PAO1Δ*algR*	PAO1 *algR* gene deleted	This study
PAO1Δ*rhlB*	PAO1 *rhlB* gene deleted	This study
PAO1Δ*lasR*	PAO1 *lasR* gene deleted	This study
PAO1Δ*lasR-rhlR*	PAO1 *lasR* and *rhlR* genes both deleted	This study
PAO1Δ*rhlR*3k	PAO1 3kb DNA fragment deleted in the *rhlR* gene locus	This study
PAO1-*trc*	PAO1 *trc* promoter insertion upstream of the *rhlA* gene	This study
PAO1-*rpsL*	PAO1 *rpsL* promoter insertion upstream of the *rhlA* gene	This study
PAO1*rhlR* W108 to stop	PAO1 *rhlR* W108 mutation to stop codon	This study
PAO1*rhlB* Q249 to stop	PAO1 *rhlB* Q249 mutation to stop codon	This study
PAK	Wild-type	(Li et al., 2013)
PAKΔ*rsaL*	PAK *rsaL*gene deleted	This study
PAKΔ*algR*	PAK *algR* gene deleted	This study
PAKΔ*lasR*	PAK *rlasR* gene deleted	This study
PAK*rhlR* W108 to stop	PAK *rhlR* W108 mutation to stop codon	This study
PAK*rhlB* Q249 to stop	PAK *rhlB* Q249 mutation to stop codon	This study
Other *Pseudomonas* spp.		
P. putida KT2440	Wild type	Lab stock
KT2440*cadR* Q92 to stop	*P. putida* KT2440 *cadR* Q92 mutation to stop codon	This study
KT2440*ompR* Q129 to stop	*P. putida* KT2440 *ompR* Q129 mutation to stop codon	This study
P. fluorescens GcM5-1A	Wild type	(Feng et al., 2015)
GcM5-1A*per* Q374 to stop	*P. fluorescens* GcM5-1A *per* Q374 mutation to stop codon	This study
GcM5-1A*aspC* Q164 to stop	*P. fluorescens* GcM5-1A *aspC* Q164 mutation to stop codon	This study
P. syringae DC3000	Wild type	Lab stock
DC3000*gacA* Q113 to stop	*P. syringae* DC3000 *gacA* Q113 mutation to stop codon	This study
DC3000*hrpL* Q29 to stop	*P. syringae* DC3000 *hrpL* Q29 mutation to stop codon	This study

Table S3. Plasmids used in this study. Related to Fig. 1, Fig. 2, Fig. 3, Fig. 4, Fig.5, and Transparent Methods.

Plasmids	Description	Reference
pAK1900	Ap^r, broad-host-range cloning vector	(Srikumar et al., 1998)
pDN19	Tc^r, *E. coli-P. aeruginosa* shuttle cloning vector	(Li et al., 2013)
pEX18Ap	Ap^r, *oriT*+ *sacB*+, gene replacement vector with MCS from pUC18	(Hoang et al., 1998)
pPS858	Ap^r, Gm^r; the vector containing gentamycin-resistance marker	(Hoang et al., 1998)
pCasSA	Km^r, Cm^r; the *S. aureus* genome editing vector	(Chen et al., 2017)
pnCasSA-BEC	Km^r, Cm^r; the *S.aureus* base editing vector	(Gu et al., 2018)
pCasPA	Tc^r, bacterial expression of Cas9 nuclease and λ-Red recombination system	This study
pACRISPR	Ap^r, a sgRNA expression plasmid for targeting a specific sequence	This study
pACRISPR-*rhlR*_spacer	pACRISPR derivative with the spacer of the *rhlR* gene	This study
pACRISPR-*rhlR*_repair	pACRISPR derivative with the repair arms of the *rhlR* gene	This study
pACRISPR-*rhlR*	pACRISPR derivative for *rhlR* deletion	This study
pACRISPR-*nalD*_spacer	pACRISPR derivative with *nalD* spacer	This study
pACRISPR-*nalD*-45bp	pACRISPR derivative for *nalD* deletion with 45bp+45bp repair	This study
pACRISPR-*nalD*-100bp	pACRISPR derivative for *nalD* deletion with 100bp+100bp repair	This study
pACRISPR-*nalD*-200bp	pACRISPR derivative for *nalD* deletion with 200bp+200bp repair	This study
pACRISPR-*nalD*-500bp	pACRISPR derivative for *nalD* deletion with 500bp+500bp repair	This study
pACRISPR-*nalD*-1kb	pACRISPR derivative for *nalD* deletion with 1kb+1kb repair	This study
pACRISPR-*rsaL*	pACRISPR derivative for *rsaL* deletion	This study
pACRISPR-*algR*	pACRISPR derivative for *algR* deletion	This study
pACRISPR-*rhlB*	pACRISPR derivative for *rhlB* deletion	This study
pACRISPR-*lasR*	pACRISPR derivative for *lasR* deletion	This study
pACRISPR-*rhlR*3k	pACRISPR derivative for deletion of 3kb fragment in *rhlR* locus	This study
pACRISPR-*rhlR*5k	pACRISPR derivative for deletion of 5kb fragment in *rhlR* locus	This study
pACRISPR-*rhlR*10k	pACRISPR derivative for deletion of 10kb fragment in *rhlR* locus	This study
pACRISPR-*lasR*-*rhlR*	pACRISPR derivative for deletion of *lasR* and rhlR genes	This study
pACRISPR-*trc*	pACRISPR derivative for *trc* promoter insertion	This study
pACRISPR-*rpsL*	pACRISPR derivative for *rpsL* promoter insertion	This study
pnCasPA-BEC	Gm^r, *Pseudomonas* spp.genome base-editing vector	This study
pnCasPA-BEC-*rhlR*sp	pnCasPA-BEC derivative with *rhlR* spacer, W108 mutation to stop codon	This study
pnCasPA-BEC-*rhlB*sp	pnCasPA-BEC derivative with *rhlB* spacer, Q249 mutation to stop codon	This study
pnCasPA-BEC-C2sp	pnCasPA-BEC derivative containing spacer with C at position 2	This study
pnCasPA-BEC-C3sp	pnCasPA-BEC derivative containing spacer with C at position 3	This study
pnCasPA-BEC-C4sp	pnCasPA-BEC derivative containing spacer with C at position 4	This study
pnCasPA-BEC-C5sp	pnCasPA-BEC derivative containing spacer with C at position 5	This study
pnCasPA-BEC-C6sp	pnCasPA-BEC derivative containing spacer with C at position 6	This study

pnCasPA-BEC-C7sp	pnCasPA-BEC derivative containing spacer with C at position 7	This study
pnCasPA-BEC-C8sp	pnCasPA-BEC derivative containing spacer with C at position 8	This study
pnCasPA-BEC-C9sp	pnCasPA-BEC derivative containing spacer with C at position 9	This study
pnCasPA-BEC-GC4	pnCasPA-BEC derivative containing spacer with GC at position 4	This study
pnCasPA-BEC-GC5AC8	pnCasPA-BEC derivative containing spacer with GC at position 5 and AC at position 8	This study
pnCasPA-BEC-GC5	pnCasPA-BEC derivative containing spacer with GC at position 5	This study
pnCasPA-BEC-AC5	pnCasPA-BEC derivative containing spacer with AC at position 5	This study
pnCasPA-BEC-*cadR*sp	pnCasPA-BEC derivative with *cadR* spacer, Q92 mutation to stop codon	This study
pnCasPA-BEC-*ompR*sp	pnCasPA-BEC derivative with *ompR* spacer, Q129 mutation to stop codon	This study
pnCasPA-BEC-*per*sp	pnCasPA-BEC derivative with *per* spacer, Q374 mutation to stop codon	This study
pnCasPA-BEC-*aspC*sp	pnCasPA-BEC derivative with *ompR* spacer, Q164 mutation to stop codon	This study
pnCasPA-BEC-*gacA*sp	pnCasPA-BEC derivative with *gacA* spacer, Q113 mutation to stop codon	This study
pnCasPA-BEC-*hrpL*sp	pnCasPA-BEC derivative with *hrpL* spacer, Q29 mutation to stop codon	This study

Ap^r, ampicillin resistant; Tc^r, tetracycline resistant; Gm^r, gentamycin resistant; Km^r, kanamycin resistant; Cm^r, chloramphenicol resistant.

REFERENCES

Brint, J.M., and Ohman, D.E. (1995). Synthesis of multiple exoproducts in *Pseudomonas aeruginosa* is under the control of RhlR-RhlI, another set of regulators in strain PAO1 with homology to the autoinducer-responsive LuxR-LuxI family. J. Bacteriol. *177*, 7155-7163.

Chen, W., Zhang, Y., Yeo, W.S., Bae, T., and Ji, Q. (2017). Rapid and efficient genome editing in *Staphylococcus aureus* by using an engineered CRISPR/Cas9 system. J. Am. Chem. Soc. *139*, 3790-3795.

Datsenko, K.A., and Wanner, B.L. (2000). One-step inactivation of chromosomal genes in *Escherichia coli* K-12 using PCR products. Proc. Natl. Acad. Sci. U. S. A. *97*, 6640-6645.

Feng, K., Li, R., Chen, Y., Zhao, B., and Yin, T. (2015). Sequencing and analysis of the *Pseudomonas fluorescens* GcM5-1A genome: a pathogen living in the surface coat of *Bursaphelenchus xylophilus*. PLoS One *10*, e0141515.

Gu, T., Zhao, S., Pi, Y., Chen, W., Chen, C., Liu, Q., Li, M., Han, D., and Ji, Q. (2018). Highly efficient base editing in *Staphylococcus aureus* using an engineered CRISPR RNA-guided cytidine deaminase. Chem. Sci. *9*, 3248-3253.

Hoang, T.T., Karkhoff-Schweizer, R.R., Kutchma, A.J., and Schweizer, H.P. (1998). A broad-host-range Flp-*FRT* recombination system for site-specific excision of chromosomally-located DNA sequences: application for isolation of unmarked *Pseudomonas aeruginosa* mutants. Gene *212*, 77-86.

Li, K., Xu, C., Jin, Y., Sun, Z., Liu, C., Shi, J., Chen, G., Chen, R., Jin, S., and Wu, W. (2013). SuhB is a regulator of multiple virulence genes and essential for pathogenesis of *Pseudomonas aeruginosa*. Mbio *4*, e00419.

Srikumar, R., Kon, T., Gotoh, N., and Poole, K. (1998). Expression of *Pseudomonas aeruginosa* multidrug efflux pumps MexA-MexB-OprM and MexC-MexD-OprJ in a multidrug-sensitive *Escherichia coli* strain. Antimicrob. Agents Chemother. *42*, 65-71.

Xie, S., Shen, B., Zhang, C., Huang, X., and Zhang, Y. (2014). sgRNAcas9: a software package for designing CRISPR sgRNA and evaluating potential off-target cleavage sites. PLoS One *9*, e100448.
